# Theta-Coupled Periodic Replay in Working Memory

**DOI:** 10.1016/j.cub.2010.01.057

**Published:** 2010-04-13

**Authors:** Lluís Fuentemilla, Will D. Penny, Nathan Cashdollar, Nico Bunzeck, Emrah Düzel

**Affiliations:** 1 Institute of Cognitive Neuroscience, University College London, London WC1N 3AR, UK; 2 Wellcome Trust Centre for Neuroimaging, University College London, London WC1N 3BG, UK; 3 Institute of Neurology, University College London, London WC1N 3BG, UK; 4 Institute of Cognitive Neurology and Dementia Research, Otto von Guericke University, 39120 Magdeburg, Germany; 5 German Centre for Neurodegenerative Diseases, Magdeburg, Otto von Guericke University, 39120 Magdeburg, Germany

## Abstract

Working memory allows information from transient events to persist as active neural representations [1] that can be used for goal-directed behaviors such as decision making and learning [2, 3]. Computational modeling based on neuronal firing patterns in animals suggests that one putative mechanism enabling working memory is periodic reactivation (henceforth termed “replay”) of the maintained information coordinated by neural oscillations at theta (4–8 Hz) and gamma (30–80 Hz) frequency [4–6]. To investigate this possibility, we trained multivariate pattern classifier decoding algorithms on oscillatory brain responses to images depicting natural scenes, recorded with high temporal resolution via magnetoencephalography. These classifiers were applied to brain activity recorded during the subsequent five second maintenance of the scenes. This decoding revealed replay during the entire maintenance interval. Replay was specific to whether an indoor or an outdoor scene was maintained and whether maintenance centered on configural associations of scene elements or just single scene elements. Replay was coordinated by the phase of theta and the amount of theta coordination was correlated with working memory performance. By confirming the predictions of a mechanistic model and linking these to behavioral performance in humans, these findings identify theta-coupled replay as a mechanism of working memory maintenance.

## Results and Discussion

**Figure 1:**
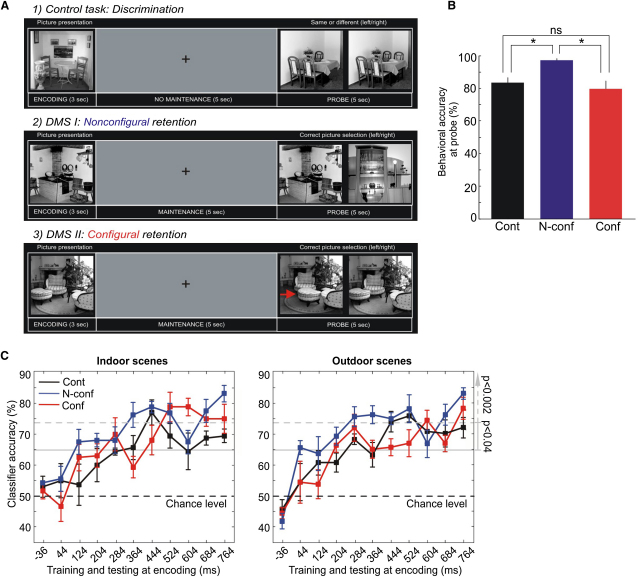
The Trial Structure, Subjects' Behavioral Performance, and MVPC Accuracy during Sample Presentation (A) Trial structure of the two variants of a blocked DMS working memory task, one with (*configural*) and the other without (*nonconfigural*) associative configural maintenance demands and a *control* task without maintenance requirements. (B) Behavioral performance at probe for each experimental condition. Working memory performance was better in the *nonconfigural* than the *configural* condition [paired t test: *t*(7) = 4.02, p = 0.005] and accuracy in *control* and *configural* was similar [paired t test: *t*(7) = 0.8, p = 0.45], showing that the two conditions were equated for difficulty. ^∗^p < 0.05; ns: p > 0.4. (C) Single-subject indoor and outdoor MVPCs were computed separately every 80 ms from −36 ms prior to 764 ms after sample onset during encoding. X axis labels time points where the MVPC was trained and tested. Plots represent subjects' mean MVPC accuracy at sample encoding for *control* (Cont; black line), *nonconfigural* (N-Conf; blue line), and *configural* (Conf; red line) conditions. MVPC results showed correct classification of sample pictures into indoor and outdoor categories from 200–300 ms onward. The statistical threshold for correct MVPC classification was set at p < 0.04 and at p < 0.002 after correcting for multiple comparisons. Error bars denote standard error of the mean (SEM) in (B) and (C).

We investigated replay under experimental conditions that allowed controlling how much associative configural information is retained during maintenance (see Figure 1A; see the Supplemental Information available online). To that end, eight healthy adults participated in two variants of a delayed match-to-sample (DMS) working memory task, one with and the other without associative configural maintenance demands. We also used a control task without maintenance requirements. In all three task conditions, the trial structure and stimulus timing were identical. In the two DMS variants, participants were presented with the image of an indoor or an outdoor scene for 3 s (all images were grayscale photographs that were normalized to the same mean gray value). This sensory input (here termed “sample”) was followed by a 5 s delay interval during which the sample had to be consciously maintained in working memory (in the two DMS conditions). After the delay, two probe stimuli, both images of scenes from the same category (indoor or outdoor), were presented side by side. Only one of the two probes (matching probe) was identical to the sample and participants were required to indicate which by a button press using the index or middle finger of the right hand. In the associative configural (henceforth called *configural*) variant of the DMS task, the nonmatching probe differed from the matching probe only in the relative location or omission of one scene element. To be able to detect the match, therefore, it was necessary to maintain a detailed record of all objects in the scene as well their associative-configural arrangement. In the *nonconfigural* condition, the nonmatching probe displayed a completely different scene. This allowed detecting the match by maintaining just one element of the sample scene. The *control* task was matched to the *configural* DMS condition in perceptual difficulty at the probe phase but did not require any maintenance of sample information. Here, both probe images were different from the sample. Subjects were instructed to indicate by button press if the two images were identical to each other and that maintaining the sample stimulus in memory would not help them to make this discrimination. *Configural* and *nonconfigural* conditions and the *control* task were separated in blocks of ten trials with four blocks each.

In summary, our methodological approach was to train individual multivariate pattern classifiers (MVPCs) to distinguish indoor from outdoor scenes separately in each of the three task conditions. These MVPCs were then used to detect replay of the sample category (indoor versus outdoor) during the delay. Specifically, we used MVPCs to test (1) whether category-selective patterns of activity elicited during sensory input would be reactivated during the delay interval, (2) whether the number of reactivations would reflect the stronger demands on maintenance in the *configural* than the *nonconfigural* condition, (3) whether these reactivations would be specific to the task condition (*configural*, *nonconfigural*, or *control*) in which the MVPC were trained, (4) whether category- and task condition-selective reactivations were modulated by theta (i.e., were more likely to occur at a particular phase of ongoing theta oscillation, i.e., were “nested”), and lastly (5) whether the number of reactivations and/or their nesting within ongoing theta correlated with the participant's ability to perform the DMS task.

MVPC training to distinguish indoor and outdoor categories of the sample was conducted every 80 ms from −36 ms prior to sample presentation to 764 ms after sample onset, independently for each time point, using the amplitudes of 38 frequencies spanning a range from 13 to 79 Hz from all 275 MEG sensors. After training, the MVPCs were successful in discriminating indoor and outdoor scenes showing similar time courses of discrimination accuracy for all three experimental conditions (Figure 1C). Successful discrimination was obtained with MVPCs trained on data acquired after the first 200 to 300 ms of sample presentation (Figure 1C).

**Figure 2:**
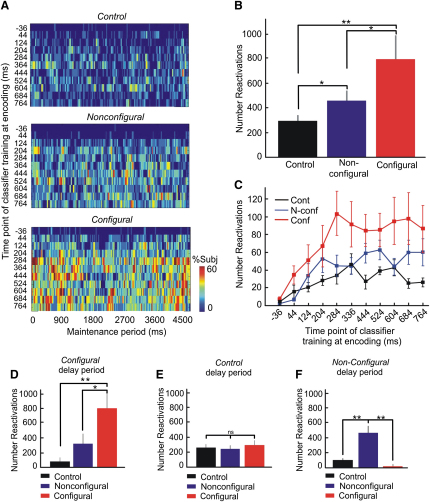
Category-, Condition-, and Task-Specific Reactivations during Maintenance (A) Category-specific replay during the maintenance period (4.5 s; x axis) for each experimental condition and for the 11 different classifiers trained at different time points of sample picture encoding (y axis). Plots represent the percentage of subjects that showed significant (p < 1.8 × 10^−5^) reactivations for different classifiers (y) and time points (x). (B) Sum of all significant reactivations for all ten (44 to 764 ms after onset of sample image) classifiers collapsed across categories and time points (paired t test one-tailed, ^∗^p < 0.05 and ^∗∗^p < 0.01). (C) Similar replay count as in (B) but displayed for each classifier. The x axis refers to each of the classifiers trained at different time points during sample picture encoding. (D)–(F) Condition specificity (*nonconfigural* versus *configural* DMS condition) and task specificity (DMS tasks versus *control* task) of reactivations. (D) Number of significant indoor/outdoor neural pattern reactivations when classifiers trained during *control* and *nonconfigural* encoding were tested along indoor/outdoor scene maintenance of the *configural* condition. This was contrasted (paired t test) with the number of significant reactivations obtained when trained and tested classifiers belonged to *configural* task. (E) As in (D), but contrasting the number of reactivations obtained during the delay of the *control* task when classifiers were trained during *configural*, *nonconfigural*, and *control* encoding. (F) As in (D), but contrasting the number of reactivations obtained during the delay of the *nonconfigural* condition when classifiers were trained during *configural*, *nonconfigural*, and *control* encoding. ^∗∗^p < 0.01; ^∗^p < 0.05; ns denotes nonsignificant. In (B)–(F), error bars denote SEM.

Using these MVPCs we observed that the category-selective activity patterns evoked by sample presentation were reactivated during the delay interval of the two DMS conditions. We defined “reactivation times” as those time points for which the classification accuracy was above a given statistical threshold (see Experimental Procedures). “Reactivations” are then defined operationally as patterns producing correct classifier outputs at reactivation times. We observed a high degree of accurate category-specific reactivation during the delay interval (Figure 2). Reactivations were observed in each participant for both stimulus categories and were distributed across the entire delay (Figure 2A), that is, they were not confined to either the early or late phases of the delay interval.

The total number of reactivations during maintenance was higher in the *configural* than the *nonconfigural* DMS condition (Figure 2B). This finding indicates that the demand to actively maintain associative configural information in working memory led to more frequent replay of information (see Figure 1B for behavioral performance in the DMS tasks). Also, both *nonconfigural* and *configural* maintenance intervals showed a greater number of reactivations when compared to those obtained during the delay period of the *control* task where no sample information needed to be maintained (Figure 2B). These findings confirm the hypothesis that maintenance in working memory is associated with replay of sensory input and show that the number of replay events increases with maintenance demands.

We then assessed whether the category-specific maintenance replay of sensory input was specific to each DMS condition. This was achieved by testing MVPCs trained during sensory input of the three task conditions on the delay intervals of the remaining two. We observed that classifier performance decreased when it was trained in another task condition (Figures 2D–2F). Fewer reactivations were found during *nonconfigural* delays when *control* or *configural* MVPCs were applied (Figure 2F). Likewise, fewer reactivations were found during *configural* delays when tested with *control* and *nonconfigural* MVPCs (Figure 2D). These findings show that, even within the same category, the content of replay was specific to each task condition. More detailed frequency-based analyses of classifier features confirmed this specificity (see Supplemental Information and Figure S3 for the spatial distribution of stable beta and gamma band features of classifiers trained in *configural* and *nonconfigural* conditions). Thus, despite the fact that the categories (indoor and outdoor) were the same and the scene images themselves were counterbalanced across each condition, participants maintained different aspects of the scenes in the *configural* and *nonconfigural* DMS conditions. This condition specificity of maintenance replay conforms to the experimental requirement to maintain associative configural information in the *configural* version as opposed to object-based information in the *nonconfigural* version. At the same time, it rules out trivial accounts of maintenance replay. Condition specificity is incompatible with the possibility that replay merely reflected some passive reverberation of the sample input that was stronger in the *configural* than the *nonconfigural* condition simply because participants were more attentive due to task difficulty.

Having identified the existence of condition-specific (*configural* versus *nonconfigural* DMS), task-specific (DMS versus *control*), and category-specific (indoor versus outdoor) reactivations during delay maintenance in working memory (see [7] for why it is appropriate to consider our DMS tasks as working memory), we then tested the hypothesis that these reactivations were periodically modulated by theta oscillations. In order to quantify the relationship between maintenance reactivations and the ongoing theta (4, 5, and 6 Hz) rhythm in the delay, we calculated the “phase-locking value” (PLV) between reactivations and theta phase [8]. This value quantifies to what extent reactivations were more likely to occur at certain phases of theta. A PLV of 1 denotes perfect phase locking with each reactivation occurring at exactly the same phase of theta, whereas a value of 0 denotes that reactivations are completely independent of the theta phase (see Experimental Procedures). For this analysis we merged the reactivations detected by MVPCs trained at different time points of sample presentation (i.e., from 44 to 764 ms after the onset of sample presentation) and calculated PLVs to theta, considering theta recordings of each of the 275 MEG sensors separately.

**Figure 3:**
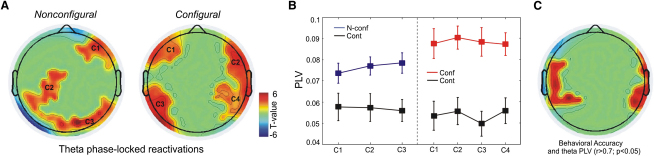
Theta Phase Coupling of Category-Specific Reactivations during Maintenance (A) Sensor-specific significant (p < 0.05) 6 Hz theta phase locking of reactivations during *nonconfigural* and *configural* maintenance. In the topographic plots, C1–C4 denote clusters (minimum of eight significant adjacent sensors) of sensors where phase locking in *nonconfigural* and *configural* conditions exceeded phase locking in the *control* task. Of these clusters, C2 and C3 in the *nonconfigural* condition and C2, C3, and C4 in the *configural* condition survived correction for multiple comparisons (see Supplemental Information and Figure S3 for details). (B) Mean 6 Hz PLVs obtained for each cluster identified in (A). Error bars denote SEM. (C) Topographic distribution of significant (red; p < 0.05, minimum of eight significant adjacent sensors) correlations between PLVs (6 Hz) at bilateral frontotemporal sensors and behavioral working memory accuracy in the *configural* condition.

Consistent with the hypothesis that maintenance replay would be modulated by theta, we found significant theta (6 Hz; see Figure S6 for PLV results at 4 and 5 Hz) phase locking for both *nonconfigural* and *configural* delay reactivations. As shown in Figure 3A, phase-locking for the *nonconfigural* and *configural* conditions engaged distinct sensor configurations. Theta phase locking for the *nonconfigural* delay reactivations was confined to frontoparietal and occipital sensor clusters (minimum of eight adjacent sensors), whereas theta phase locking of *configural* delay reactivations occurred over bilateral frontotemporal sensor clusters (for the identification of sensor clusters see Supplemental Information and Figure S4). Furthermore, in both the *nonconfigural* and *configural* conditions, sensors from significant clusters showed higher 6 Hz theta PLVs when compared to the *control* task (Figure 3B). This was also confirmed by a within subject and condition permutation analysis (see Supplemental Information and Figure S5). This topographic separation indicates that *nonconfigural* and *configural* replay was modulated by different theta networks: a frontoparietal and occipital theta network modulated replay of *nonconfigural* information, whereas a frontotemporal theta network modulated *configural* replay (Figure 3A).

Having shown that category- and condition-specific replay during maintenance was periodically modulated by different theta networks, we then assessed to what extent the number of reactivations and theta modulation of replay were correlated with working memory performance. As expected, the *configural* DMS was more difficult than the *nonconfigural* DMS condition [mean rate of identifying the correct match: *nonconfigural*: 97%, and *configural*: 79%; *t*(7) = 4.01, p < 0.01; Figure 1B], whereas there was no performance difference between the *control* task (mean rate of detecting whether the two probes were the same or not: 83%) and *configural* DMS [*t*(7) < 1], showing that we were successful in matching the difficulty between the *configural* DMS and the *control* task. We observed a positive correlation (Pearson's coefficient, *r* > 0.7; p < 0.05, two-tailed, minimum of eight significant adjacent sensors) between frontotemporal sensors theta PLVs and the accuracy in correctly identifying the matching probe in the *configural* condition (Figure 3C). Since both probes were from the same category, this correlation argues against the possibility that participants merely maintained a “semantic label” of the sample category (i.e., indoor/outdoor). The total number of category reactivations during the delay was not correlated with working memory accuracy (all sensors p > 0.05). A correlation analysis was not performed for the *nonconfigural* condition because working memory performance was nearly perfect (mean 97%, SD 0.2%). These results indicate that associative configural working memory performance depends on the clocking and coordination of periodic replay by ongoing theta dynamics rather than the absolute number of reactivations.

Our findings provide evidence for the prediction from animal physiology [9] and computational modeling [4, 6] that working memory maintenance in humans is associated with periodic replay of sensory input and that the periodicity of replay is modulated by slow oscillatory rhythms in the theta frequency range. Indeed, in animals it is meanwhile well established that theta phase can modulate the stimulus-specific spiking of neural ensembles in many different brain regions, such as the hippocampus, visual cortex, and prefrontal cortex [9–13]. During spatial exploration, the theta rhythm modulates the activity of rodent hippocampal [11, 14, 15] and entorhinal [16, 17] place-coding neurons in a phase-dependent manner. In nonhuman primates, neuronal spiking in visual area V4 is modulated by theta phase during delay maintenance [9]. Recently, invasive recordings in humans showed that fast oscillatory rhythms, which presumably code stimulus-specific information, are also “nested” within theta phases [18].

Theta-coupled periodic replay is likely to interact [19] with another neural mechanism of working memory that cannot be directly measured with MEG, namely, the persistence of neural firing from stimulus processing into maintenance. Although persistent neural firing during delay maintenance has been observed in many different brain regions, including medial temporal, prefrontal, and parietal regions [20, 21], in some regions, such as area V4, neural delay firing is largely periodic and theta coupled [9]. Despite such anatomical separation, it is physiologically plausible that persistent firing and theta periodic activity could functionally interact to sustain each other [19, 22]. Through such interaction, persistent firing coding multimodal stimulus attributes [22] or task-related information such as goals and cognitive control signals could sustain periodic replay of stimulus-specific information. Indeed, an influential model of working memory in humans [1] postulates a rehearsal mechanism (which we see as being related to replay) controlled by the central executive. The *nonconfigural* version of our working memory task has the closest correspondence to a working memory study in nonhuman primates demonstrating theta-coupled replay in visual area V4 [9]. We observed quite widespread activity in this task involving not only the occipital MEG sensors but also frontal and parietal sensor regions (Figure 3A and Figure S6). This widespread distribution suggests that persistent firing and periodic replay may have the opportunity to interact locally within frontal and parietal regions.

Recent animal recordings suggest that the hippocampus may actively control the transfer of cortical information to the hippocampus itself via theta-phase biasing of neocortical network dynamics [13]. On the basis of the MEG sensor data reported here, we cannot separate such hippocampal theta entrainment from a cortico-cortical theta entrainment (for review see [23, 24]) during periodic replay. We also cannot exclude the possibility that hippocampal theta generation has directly contributed to our measures.

The possibility of investigating periodic replay noninvasively and at high temporal resolution opens new perspectives for uncovering the neural mechanisms underlying cognitive processes where chronometry is a critical factor [4, 6]. Given the distributed nature of the neural populations that are likely to contribute to replay, high-density whole-head recordings (by MEG or EEG) with their very large sampling space seem ideal for this purpose in humans. Focal, anatomically targeted recording techniques, such as intracranial recordings, offer insight into how single neurons and small neuronal populations are entrained into network behavior but the clinical constraints on these recordings and their limited sampling space should make it challenging to detect replay in humans. Hence, the combination of high density MEG/EEG with focal intracranial recordings seems particularly suited to understand how replay is coordinated with local neuronal ensembles.

By decoding working memory content at high temporal resolution we confirm the core prediction based on animal physiology [9, 15] and a model based on animal physiology [6] that theta-coupled periodic replay serves as a neural mechanism underlying the maintenance of information in human working memory. This holds particularly for associative configural working memory where theta phase coupling during maintenance correlated with working memory performance. These results help to narrow a long-standing gap between memory research in humans and data and models based on the temporally fine grained dynamics of memory mechanisms identified in animals.

## Experimental Procedures

### Subjects

Eight right-handed healthy subjects (two female; mean age 21, SD 1.3) participated in the experiment after giving written informed consent. The study was approved by the University of London Research Ethics Committee for human-based research. All participants were financially compensated for their participation.

### MEG Recordings

MEG data was recorded with a 275 channel CTF Omega whole-head gradiometer system (VSM MedTech, Coquitlam, BC, Canada) with a 480 Hz sampling rate and 120 Hz low-pass filtering. After participants were comfortably seated in the MEG, head localizer coils were attached to the nasion and 1 cm anterior of the left and right tragus to monitor head movement during the recording sessions.

### Single-Trial Time-Frequency Analysis

Data were analyzed offline with Matlab v7.1 (Mathworks, Natick, MA). Epochs of 9 s, including a 1000 ms baseline preceding the onset of sample presentation, 3000 ms of sample presentation, and 5000 ms of maintenance period, were used in the time-frequency (TF) analysis. The 1000 ms preceding sample onset and following the end of the maintenance period were included in order to avoid edge-effects in the subsequent wavelet analysis. Data were downsampled to 250 Hz after TF analysis. TF was computed by a continuous wavelet transformation (CWT) on single-trial data for each subject and sensor via a complex Morlet wavelet defined as:

$$w\left(f,t\right)={\left(2\pi \sigma_{t}^{2}\right)}^{-1/2}e^{\frac{-t^{2}}{2\sigma_{t}^{2}}}e^{2i\pi f_{0}t},$$

where the relation *f_0_/σ_f_* (where *σ_f_* = 1/(2*πσ_t_*) was set at 7 [25]. The TF representation of the signal *s*(*t*), trial *k*, frequency *f*, and time *t* was computed as

$$F_{k}\left(f,t\right)=w\left(t,f\right)\times s_{k}\left(t\right),$$

where *x* denotes the complex convolution. Frequencies were selected in steps of 1 Hz within the 2–20 Hz frequency range and in steps of 2 Hz within the 21–79 Hz frequency range. For every time window and frequency bin, instantaneous spectral amplitude was computed by taking the modulus of the resulting CWT coefficient, squaring and adding them, and then taking the square root (i.e., for each time and frequency bin). Spectral amplitude data were then normalized at the single-trial level by subtracting mean spectral amplitude during the baseline period, defined as 500 to 100 ms prior to picture onset at the sample.

### MEG Multivariate Pattern Classification Analysis

MVPC analysis was implemented with the Matlab Neural Network Toolbox (Mathworks) and some of the software routines available from the Princeton Multi-Voxel Pattern Analysis for fMRI website (http://www.csbmb.princeton.edu/mvpa) but modified and adapted to MEG data.

We used univariate statistics at each sensor and TF bin in order to select those features that would constitute the independent variables (i.e., the inputs) for the classifier (feature selection). Those features (i.e., spectral amplitude at particular TF bins and sensors), which were found to be significantly different between categories (using n = 20 indoor and n = 20 outdoor exemplars) by a two-tailed paired Student's t test (p < 0.05), were selected. This data-led process served to reduce the dimension of the pattern classification problem. This feature selection process was repeated for classifiers trained on data from different time points during sample encoding (see below)—that is, classifiers trained at different times in the encoding interval used different features.

Low-frequency components (2–12 Hz) were not used to train MVPCs. This reasoning was based on two arguments. First, we assumed that low frequency components were not needed for capturing neural representations of visual category-specific information. Indeed, previous studies [26] suggest that higher frequency M/EEG components in the gamma range (i.e., 31–79 Hz) can represent specific object properties (see Figure S3). Second, we wished to investigate whether these representations were replayed in short-term memory through a patterned reactivation process that is phase coupled with the ongoing theta rhythm [4, 27].

We trained 11 separate classifiers from data at −36, 44, 124, 204, 284, 236, 444, 524, 604, 684, and 764 ms relative to the onset of sample presentation. Given that data from the baseline interval (−36 ms) did not contain any category-specific visual stimulation, we hypothesized that pattern classifier prediction accuracy should be at the chance level (i.e., 0.5 for two categories) for all the experimental conditions. Each subject viewed 20 indoor and 20 outdoor pictures per experimental condition during encoding. Each of the 11 classifiers was trained with data from each experimental condition separately. This yielded 40 training patterns (20 per category) for each of the 11 classifiers (n = 11) and for each experimental condition (*control*, *nonconfigural*, and *configural*). Indoor and outdoor information across conditions was not grouped to a single classifier training session in order to assess possible differences related to sample encoding during the three experimental conditions. Here, neural network optimization (i.e., learning) was based on the conjugate gradient algorithm (“traincgb” in Matlab) [28]. Neural network topology was defined by an input layer, which contained each of the sensor/frequency features, a hidden layer comprising 20 units, and an output layer, defined by two units, one for each of the category-specific patterns in our study (indoor and outdoor scenes). The target patterns were (1 0) for an indoor scene and (0 1) for an outdoor scene. Neural network training was always stopped after 20 iterations.

We then applied (i.e., tested) these trained MVPCs to (1) MEG responses elicited by different exemplars at the same encoding time point and (2) during the maintenance interval.

First, we determined the development of category-specific (indoor/outdoor scenes) neuronal representations during the encoding period. This was implemented with a cross-validation process. Cross-validation is the statistical practice of partitioning a sample of data into subsets such that the analysis is initially performed on a single subset (training set), while the other subset (testing set) is retained for subsequent use in confirming and validating the initial analysis. For each classifier we used the leave-one-out cross-validation (LOOCV) method to obtain an unbiased estimate of classification accuracy [28]. In LOOCV, a single observation (i.e., a trial) is first removed from the original data set and a model is built on the remaining observations (i.e., n − 1 *trials*). Subsequently, the model is used to predict the response for the held-out observation. This process is then repeated for each remaining observation and prediction accuracy is averaged over the held-out observations. In each LOOCV-iteration test data and training data are strictly separated. In a second step, we used trained classifiers to test, at single-trial level, whether TF data of the maintenance interval could be accurately classified as indoor versus outdoor. For these analyses, a cross-validation procedure is not required as the exemplars during the maintenance interval are independent of those used to train the classifiers.

For a given trained classifier, we tested at 250 consecutive time points of the maintenance interval (corresponding to 4.5 s after excluding the first and the last 250 ms of the maintenance interval) whether the trained classifiers could discriminate between indoor/outdoor scene maintenance based on selected TF features at that time point. At each time point on each trial the classifier outputs were then thresholded [by using a value of >0.95 from a possible output value range of 0 to 1 (perfect discrimination)]. In other words, if the relevant output was >0.95 the classification was deemed correct, e.g., (0.96 0.31) for correct indoor and (0.42 0.98) for correct outdoor. We based our decision criteria on a probability function to consider only correct category outputs during the maintenance period [see Figure S1 for an analysis of the degree to which a classifier could estimate (again by using a threshold classifier output of >0.95) that neural activity for a particular trial and time bin could be simultaneously classified into both categories]. The resulting thresholded output was then set to y = 1 for a correct output and y = 0 for an incorrect output. We then assessed within task condition (*control*, *nonconfigural*, and *configural*) classifier accuracy over trials for each subject. This was computed separately for each scene category (indoor/outdoor). If *C* is the number of trials correctly classified then P(*C*) follows a binomial distribution with correct probability *r* and n = 20 (we have 20 observations of each trial type). We tested against the null hypotheses of classification at the chance level (*r* = 0.5) by using the normal approximation to the binomial density, which allowed us to compute p values. A p value of 0.01 for example corresponds to ∼15/20 correct trials. We then applied a correction for multiple comparisons (over the 250 time points). A corrected p value of 0.05 was then obtainable with a threshold of p = 1.8 × 10^−5^ (i.e., 250 time points × 11 classifiers). This corresponded to 20/20 correct trials (i.e., “perfect” classification accuracy). We then defined reactivation times as those time points for which the classification accuracy reached this threshold. Reactivations are then defined operationally as patterns producing correct classifier outputs at reactivation times.

We then counted the number of reactivations for classifiers trained at different points during the encoding interval and computed the total. This total number of reactivations was computed separately for each task condition, category, and subject.

### Theta Phase Coupling of Category-Specific Reactivations

For quantification of the degree to which reactivations during maintenance were phase coupled to the ongoing theta rhythm, single-trial data were high-pass filtered at 3 Hz with a zero-phase filter (“filtfilt” in Matlab), and then instantaneous theta phase was estimated for each time point and sensor. During CWT, a normalization factor assured that a signal with a maximum amplitude of 1 resulted in a transform with maximum amplitude of 1. The modulus of the resulting TF coefficient matrix denotes absolute amplitude, whereas the inverse tangent of its imaginary-to-real part ratio denotes phase. Instantaneous phase information was calculated separately for 4, 5, and 6 Hz.

Phase coupling of reactivations to a given theta oscillation implies a phase concentration of reactivation times. We therefore computed, for each sensor, the degree of phase alignment of the given phases. Phase alignment for each trial *j* was measured with the PLV of Tallon-Baudry et al. [8] as

$$PLV_{j}=\frac{1}{N_{j}}\left|\sum_{n=1}^{N_{j}}\exp\left(i\phi_{nj}\right)\right|,$$

where $\phi_{nj}$ is the theta phase at the *n*th reactivation on the *j*th trial and || represents the complex norm. A value of 1 would correspond to perfect phase alignment and a value of 0 to uniform phase distribution across time points (see Supplemental Information for a discussion of why PLV values should not be affected by the reactivation criterion mentioned above).

The PLVs were then *z* normalized by the use of the arcsine transform [29] as

$$z_{j}^{p}=\sin^{-1}\left(2PLV_{j}-1\right)\text{.}$$

The normalized *z* variates were then used to make comparisons between conditions at each sensor. First, we used paired Student's t tests to see whether PLVs differed significantly between DMS task trials (*configural* or *nonconfigural*) and *control* task trials. The *control* delay period provides a good “baseline” condition because the physical characteristics of the visual stimulation (i.e., fixation cross) are identical to those during DMS maintenance. While subjects were required to maintain a memory of the sample stimulus during the delay period in the *nonconfigural* and *configural* task conditions, there were no working memory requirements during the *control* delay period. Those sensors that showed significant (p < 0.05) differences during the t test analysis were brought to a cluster-based nonparametric permutation test to deal with the multiple comparisons problem (see Supplemental Information for details). Finally, we also conducted a within-subject permutation analysis to estimate the probability of observing significant phase locking (between theta and reactivations) based on the true temporal correlation structure of the reactivation vectors (see Supplemental Information for details and Figure S5 for results).

## Supplemental Information

Document S1. Supplemental Experimental Procedures, Supplemental Results, and Seven Figures
