# Supplementary material for: Theta-Coupled Periodic Replay in Working Memory
Source: Curr Biol. 2010 Apr 13;20(7):606–12. doi: 10.1016/j.cub.2010.01.057 (PMC2856918; doi:10.1016/j.cub.2010.01.057)
Supplement: Document S1. Supplemental Experimental Procedures, Supplemental Results, and Seven Figures [file mmc1.pdf]

Current Biology, Volume *20*

Supplemental Information

Theta Coupled Periodic Replay in Working Memory

Lluís Fuentemilla, Will D. Penny, Nathan Cashdollar, Nico Bunzeck, and Emrah Düzel

1. Supplemental Experimental Procedures

1.1. Stimuli and task design

The stimuli used in this experiment were photographs of 260 indoor and 260 outdoor scenes. All pictures comprised 300x300 pixels. They were grey scaled (8 bits; range 0-255), normalized to a mean grey value of 127 (SD 75) and superimposed upon a grey background (mean grey value of 127). The experiment consisted of two types of tasks; a Delayed-Match-to-Sample (DMS) and a *control* task. The DMS task had a *nonconfigural* and a *configural* condition. In all three task conditions, the trial structure and stimulus-timing were identical. After a 2 sec inter-trial interval, an indoor or an outdoor scene (sample) was presented for 3 sec. This was followed by a blank screen with a fixation cross for 5 sec (delay period) and then by two test stimuli (probes) for 5 seconds (Figure 1A).

Retention of the sample stimulus across the 5 second delay period was necessary only in the two DMS conditions. In the DMS task, the two test images after the delay interval were used to probe memory for the sample. In the *nonconfigural* DMS condition, one of the probes was an exact repetition of the sample and the other depicted an entirely different novel scene of the same category (indoor or outdoor) as the sample. In the *configural* DMS condition, the non-matching probe stimuli were very similar to the sample. This was achieved as in earlier studies [1] by manipulating the relations among some elements of the scenes through (i) addition or deletion of a new object (56% of all trials) or (ii) a spatial shifting of an object within a scene (44 % of all trials). For example, in Figure 1A, the non-matching probe image (left) shows that location of the sofa is changed. In the *nonconfigural* variant, the non-matching stimulus depicted a completely different scene. Hence, the *nonconfigural* variant could be solved by maintaining non-associative object-based information about the sample across the delay interval, while the *configural* variant could only be solved by maintaining a detailed image of all objects in the scenes as well as their *configural* (spatial) associative relationship to each other. In both versions of the DMS task, participants were instructed to maintain the sample image in their memory in order to be able to correctly identify the

matching sample. The location (left or right) of the matching and the non-matching probes were counterbalanced across trials.

In the test phase of the *control* task, two scene images, both of which were different from the sample, were presented side-by-side and subjects were instructed to indicate if the two pictures were the same or different. In half of the trials the pictures were identical and in the other half they differed to the same degree as the probes in the *configural* DMS condition. This was achieved by carefully counter-balanced assignment of the same set of images to different experimental tasks across subjects. Subjects were instructed that they should not maintain the sample stimulus in memory as this would not help them to make the difficult same/different discrimination during the test phase. The *control* task was designed in a way to match the difficulty of the *configural* DMS condition in perceptual decision at probe presentation as well as to account for anticipatory neural delay activation in both DMS conditions.

Configural, *nonconfigural* and *control* conditions were separated in blocks of 10 trials each. Subjects were instructed prior to each block as to which task condition would be tested. There were 4 blocks each, resulting in 40 trials per task condition. The presentation of indoor/outdoor stimuli was counterbalanced across each block and was kept constant across individual trials. There was no response deadline within the 5 second period of probe presentation and responses were given using button presses with the index and middle finger of the right hand.

In order to ensure that participants attentive to the content of each sample during encoding, $1/10^{th}$ of trials were so-called “catch trials”. Here, grey scaled images depicting male/female faces were presented (these images were normalized in the same manner as the indoor/outdoor images). Subjects were required to respond (by button press) with their right index finger to each catch trial. A catch trial was followed by a blank period and two empty side-by-side squares were presented instead of pictures at probe.

1.2. Cluster-based nonparametric permutation test

We used an MEG-based nonparametric permutation test described by Maris and Oostenveld [2,3] to deal with the multiple comparisons problem (MCP) given by the large number of sensors (275). This method provides three important advantages: (1) it provides a simple way to solve the MCP, (2) it allows incorporating prior knowledge about the type of effect that can be expected and (3) it does not depend on parametric assumptions about the probability distribution of the data.

The analysis was implemented to compare the PLVs in each of the two memory conditions (*nonconfigural* and *configural*) with the *control* task. We first selected all sensors whose paired *t* test value (e.g. *configural versus control*) was larger than the threshold of

$p<0.05$ (uncorrected). We then clustered the selected sensors into connected sets on the basis of spatial adjacency, defined by a criterion of a minimum of 8 adjacent sensors. The permutation distribution was then obtained by: (1) collecting the trials of the two relevant experimental task conditions (e.g. *configural and control*) in a single set, (2) randomly partitioning the trials into two subsets, (3) for each cluster, computing the sum of the *t*-values, and then taking a test statistic equal to the maximum of the cluster-level statistic and (4) repeating steps 2 and 3 1000 times to construct a histogram. The nonparametric statistical test was finally performed by calculating a "Monte Carlo" p-value under the permutation distribution and comparing it with an alpha-level of 0.05.

1.3. Within-subject theta phase-coupling of reactivations

Note that reactivation time points were defined by perfect classification across all 20 trials of one category and task type. This means that reactivation time points represent a subsample of the "true" within-trial distribution of correct classifications. For the phase-locking analysis, it is not relevant whether this subsample is sparse (because the necessary temporal alignment across trials occurs rarely), or is close to the 'true' number of reactivations (because there is strong temporal alignment across trials). In both cases, reactivation time points should represent the phase-distribution of "true" within trial correct classification and are therefore suitable to assess phase-coupling to theta.

To assess whether reactivations were significantly coupled to theta-phase, we constructed a null distribution separately for each task type (*nonconfigural* and *configural*). For each individual subject we randomly shifted (or rotated) the theta-phase vectors of the 40 individual trials belonging to one task type without changing the ordering within the reactivation vector corresponding to the same task type.

Hence, theta (6Hz) phase of the *j*th trial was perturbated randomly by ɵj (ɵj is drawn from a uniform distribution on {0,2pi}). This means that for all time points (starting from 250 ms after the beginning of the delay period to 250 ms before the end of the delay period) the same phase shift was applied. After each phase shift we computed a single PLV from all 40 trials of a given task type, using data points from the delay period. This was repeated for each sensor over 1000 permutations and the resulting 1000 PLVs were *z*-normalized to create an empirical distribution. These steps were first computed separately for each of the 10 classifiers from the encoding period and the resulting distributions from the 10 classifiers were then averaged. This averaged distribution provides an estimate of the confidence limits on the observed *z*-PLVs (averaged over classifiers) at each sensor and for each individual task type and subject. Those sensors for which this p-value exceeded an alpha-level of 0.05 were deemed to contain significant theta phase-coupling of reactivations. Note that this procedure is suitable to test the reliability of theta-phase coupling of reactivations without removing the temporal

correlations present within reactivation vectors. This analysis controls for within-trial temporal correlations. If the reactivation phases are consistent between trials we should see a significantly non-zero PLV.

We additionally note that the phase-locking analysis in sensor space may be confounded by the fact that the same neuronal sources may give rise to activity at multiple diffuse sensors. This is only a problem, however, when computing PLVs between sensors. In our analysis we computed PLVs of reactivation times within each sensor.

2. Supplemental Results

2.1. Pattern classification accuracy at encoding

The accuracy of the pattern classifier during encoding was subjected to a repeated measures ANOVA including the following within-subject factors: Category (2 levels: Indoor and Outdoor), Task condition (3 levels: *control*, *nonconfigural* and *configural*) and Time (11 levels corresponding to the 11 classifier time points used). Across time, pattern classification estimates evolved similarly across conditions, that is after chance level accuracy (i.e. ~50%) during pre-stimulus and early stimulus onset periods, all *control*, *nonconfigural* and *configural* outcomes increased to 70-80% at around 200 ms post-stimulus onset (Time: $F[10,70]=28.87$; $p<0.001$; Task condition x Time: $F[20,40]<1.51$). No main effects were found for classifier accuracy between indoor and outdoor scenes (Category: $F[1,7]<0.2$), allowing us to infer that neural network classifiers had similar prediction accuracy across both categories.

2.2. Gamma band activity (31-79 Hz) and category-specific neural representation

The role of gamma band activity in object representation was also investigated [4]. Independent pattern classifiers were trained and tested during encoding (at -36, 44, 124, 204, 284, 236, 444, 524, 604, 684 and 764 ms post-stimulus onset) but only with indoor/outdoor category features (i.e. sensor/frequency wise) selected from the gamma range (31-79 Hz). The steps of this analysis exactly followed those performed previously with the exception of a wider frequency range (13-79 Hz). In a second step, the same analysis was repeated but including only features selected from lower frequencies, namely those in the 13-29 Hz range.

Networks trained only from gamma band activity correctly predicted image category (indoor versus outdoor) (Figure S2) and classifier accuracy was higher for the gamma band features when compared to the 13-28 Hz range (repeated measures ANOVA; $F[1,9]=5.06$; $p<0.06$).

Finding correct classification based only on gamma band activity opens the possibility of further increasing the temporal resolution with which reactivation patterns can be detected. However, it should be noted that there is a trade-off between frequency and time resolution in our analysis based on continuous wavelet transformation of the data (i.e., uncertainty principle). That is, given a signal with some event in it, one cannot assign simultaneously an exact time and frequency response scale to that event. The product of the uncertainties of time and frequency response scale has a lower bound. Thus, in the scaleogram of a continuous wavelet transform of this signal, such an event marks an entire region in the time-scale plane, instead of just one point. Due to this trade-off, further improving the temporal resolution of MVPC may diminish classifier performance because of a decrease in frequency resolution. The meaningful boundary conditions of this time-frequency trade-off for this type of analysis remain to be fully investigated.

2.3. Spatial characteristics of the reactivation patterns

In order to assess the stable contributions of different frequency bands in the different task conditions we determined the spatial characteristics of classifier features leading to the detection of reactivation patterns. This was done by selecting those frequency/sensor classifier features that were identified during the feature selection step of the MVPC analysis (*t* tests). For each of the 10 classifiers trained after stimulus onset, features were grouped into frequency bands, namely, low beta (13-15 Hz), high beta (18-29 Hz), low gamma (31-45 Hz) and high gamma (55-79 Hz). Using SPM5 (Wellcome Trust Centre for Neuroimaging, University College London, UK) individual maps for each frequency band and each of the 10 encoding time points were converted into NIFTI format. Each point in these maps corresponded to the number of frequency bins, within the relevant frequency band, that were chosen in the feature selection step.

Channel space maps were then created by projecting the sensor locations onto a plane followed by a linear interpolation to a 64x64 pixel grid (pixel size = 3x3mm). We performed one-sample *t* tests for all subjects to identify significant voxels within each map ($p=0.005$, uncorrected).

We then sought to identify which classifier features remained stable across the 10 encoding time points by performing a conjunction analysis, thresholded at $p=0.05$ (uncorrected), on the resulting significant voxels from each time point (Figure S3).

This conjunction analysis revealed that some features in fact did remain stable across the 10 encoding time-points. For instance, the contribution of low beta oscillations to classifier features remained stable in a frontoparietal sensor distribution in both the *nonconfigural* and *configural* conditions, while showing no stability in the *control* task. Remarkably, differences were found between *nonconfigural* and *configural* conditions in the stability of high beta, low

and high gamma bands. In the *nonconfigural* condition, stable features emerged mainly in the high beta band with a frontoparietal and bilateral temporal distribution. In the *configural* condition, on the other hand, stable features emerged for the low and in a more pronounced fashion for the high gamma band over bilateral frontotemporal regions and extending to parietal and occipital regions. Interestingly, the spatial distribution of the stable high-gamma band classifier features in the *configural* condition was remarkably similar to the spatial distribution of phase-locking between theta and reactivations during the delay (Figure 3A in the main manuscript).

2.4. Theta phase-coupling of reactivations

Cluster-based permutation results

Results of the cluster-based nonparametric permutation test over those sensors showing significant ($p<0.05$, uncorrected) theta phase-coupling of reactivations when comparing the memory tasks (i.e., *nonconfigural* and *configural*) to the *control* task revealed the existence of different clusters of sensors that were above the defined significance level ($p<0.05$, corrected), as estimated from the permutation distribution.

Figure S4 summarizes these results. Three main clusters were identified when comparing theta PLVs in *nonconfigural* to *control* task conditions (Figure S4B); right frontal (cluster 1, $p=0.2$, corrected), left parietal (cluster 2, $p=0.001$, corrected) and right parieto-occipital (cluster 3, $p=0.04$, corrected). Only the latter two clusters were significant after correcting for multiple comparisons. For the *configural* versus *control* task conditions (Figure S4C) we found four clusters; left frontal (cluster 1, $p=0.3$, corrected), right temporal (cluster 2, $p=0.02$, corrected), left temporal (cluster 3, $p=0.01$, corrected) and right frontotemporal (cluster 4, $p=0.02$, corrected). Only the latter three clusters were significant after correcting for multiple comparisons.

Within-subject theta phase-locking

Sensors identified by the cluster based permutation test were considered for a permutation analysis to determine whether theta phase-locking of reactivations within DMS conditions exceeded chance level. This showed significant ($p<0.05$) theta phase-locking for individual subjects in the *nonconfigural* and *configural* DMS conditions for most of the sensors within clusters (Figure S5).

Reactivation times versus correct classifications within trials

We defined reactivation times during the delay period as correct classifier outputs (above 0.95) that consistently occurred in each trial. Using this criterion, the mean number of reactivations across all time points of the delay period and all 10 classifiers from the encoding

period was 399 (per category) for the *configural* task. In contrast, when considering correct classifier outputs (summing all time points where any of the 10 classifiers outputs from the encoding period was above 0.95) independently for each trial, the mean number of correct classification per trial was 1384 (per category) in the *configural* task. Therefore, this shows that, as expected, reactivations times reflected a subsample of correct classifier outputs. Future studies may provide statistical approaches to utilize correct classifier outputs within single trials to assess reactivations.

Theta phase-reset during the delay interval

Theta (6Hz) phase reset was observed at the onset of the *configural* condition delay maintenance period for those sensor clusters that survived correction for multiple comparisons in the previous analysis (see *Cluster-based permutation results*) (Figure S7). This strong theta phase-reset indicates that oscillatory activity was phase aligned at the beginning and over frontotemporal sensors also at the end of the delay period. This phase alignment may account for the higher proportion of reactivations detected in the first and last sections of the delay period.

Supplemental References

1. Ryan J.D. and Cohen N.J. (2004). Processing and short-term retention of relational information in amnesia**.** Neuropsychologia *42***:** 497-511.

2. Maris E. and Oostenveld R. (2007). Nonparametric statistical testing of EEG- and MEG-data**.** J. Neurosci. Methods *164***:** 177-190.

3. Maris E., Schoffelen J.M., and Fries P. (2007). Nonparametric statistical testing of coherence differences**.** J. Neurosci. Methods *163***:** 161-175.

4. Tallon-Baudry C. and Bertrand O. (1999). Oscillatory gamma activity in humans and its role in object representation**.** Trends Cogn Sci. *3***:** 151-162.

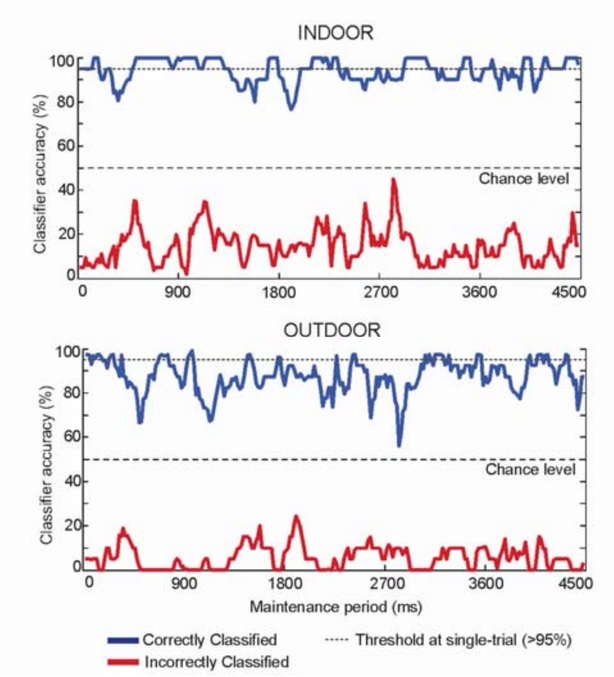

Figure S1. Correct and incorrect MVPC outputs during delay periods from 20 *configural* DMS trials of a single subject. MVPC classifications were based on the two neural network outputs, y1 for indoor and y2 for outdoor, at each time point and trial. For trials that were truly indoor (i.e., an indoor picture was to be maintained during delay interval), MEG patterns were "correctly" classified as indoor if y1>0.95. Similarly, for outdoor patterns (y2>0.95). The figure shows the resulting probabilities for correct (blue) and incorrect (red) classification, as computed across 20 trials of one subject, for each image category as a function of time in the delay. These illustrative results were obtained for a single classifier (trained at 284 ms at encoding) from the *configural* condition. Note that, each neural network output (e.g. y1) provides two probability outcomes. One refers to the likelihood that data from trial *j* at time point *t* belongs to y1 and a second one provides the probability that this same trial *j* and time point *t* does not belong to y1. Therefore, the sum of the probabilities to the correct and incorrect classifier to y1 at trial *j* and time point *t* should always equal 1. This means that the probability of an incorrect output to y1 equals 1 minus the probability of a correct output to y2 and vice versa. Note that these results in correct output being mirror images of incorrect outputs for the other category.

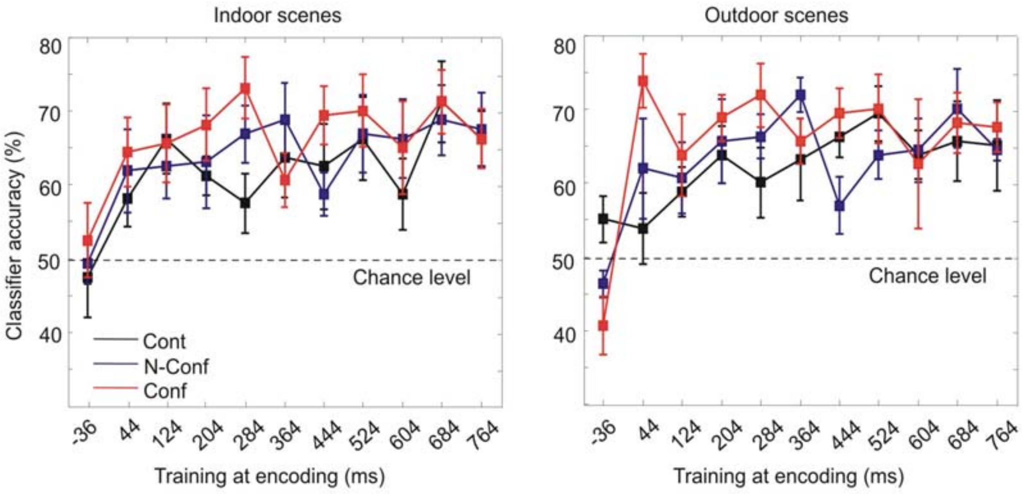

Figure S2. MVPC accuracies obtained for indoor and outdoor scenes at encoding for *control* (Cont), *nonconfigural* (N-Conf) and *configural* (Conf) task conditions when trained and tested only with gamma band (31-79 Hz) amplitude data. Error bars denote standard error of the mean.

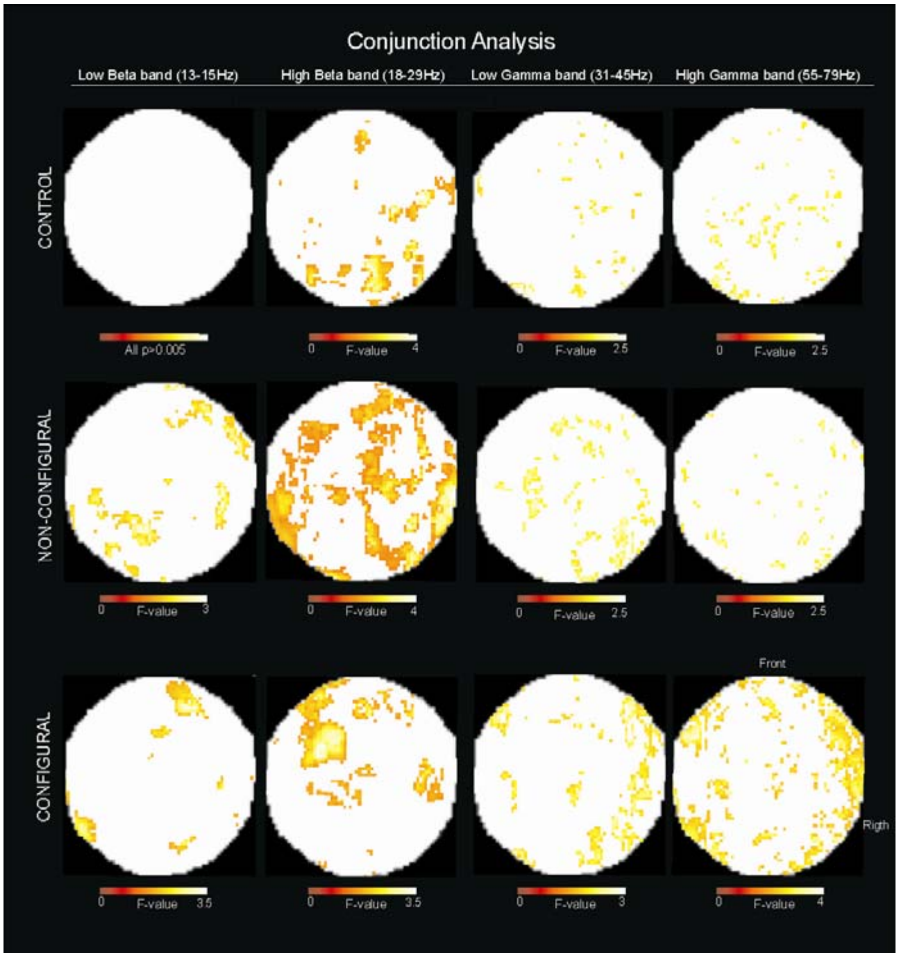

Figure S3. Topography of MVPC features that were consistent across the 10 classifiers trained after the onset of sample presentation during encoding. Note the consistent contribution of high-gamma band oscillations in the *configural* DMS task. These results indicate that high-gamma band MVPC features were strongly redundant across the classifiers trained at different time points after the onset of sample presentation. Note that colour scales represent different F-values between plots.

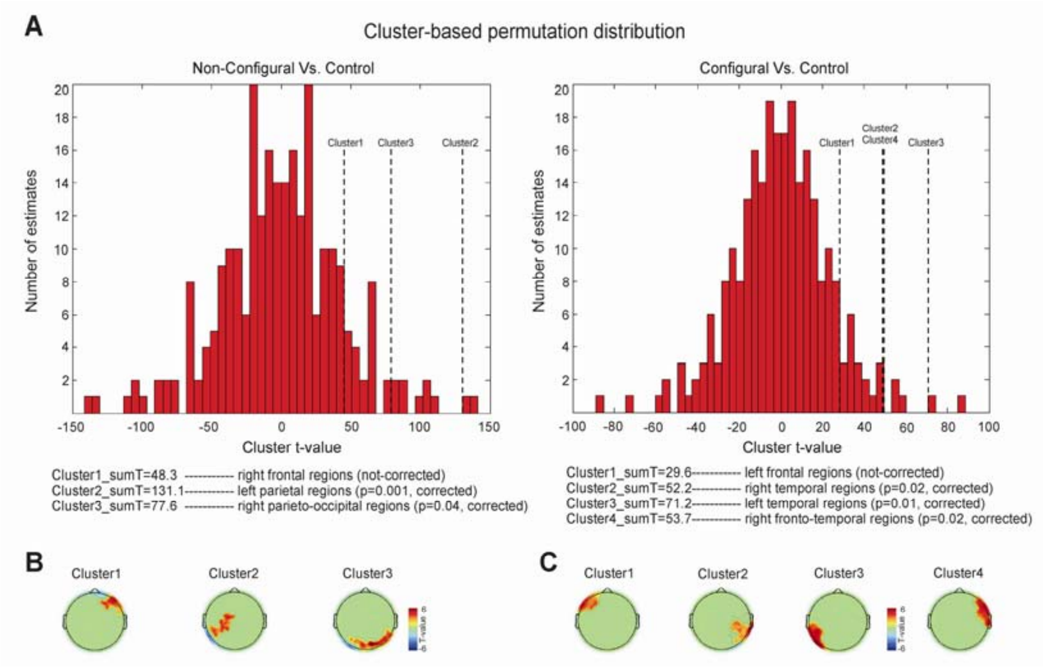

Figure S4. Results of the cluster-based nonparametric permutation test. (**A**) Distribution of max summed t-values for clusters when concatenating real 6 Hz PLV data from *nonconfigural* and *control* (left) and *configural* and *control* (right) maintenance periods. (**B**) Topography of clusters that were identified based on sensor adjacency for the *nonconfigural* condition. (**C**) Same as **B** for the configural condition.

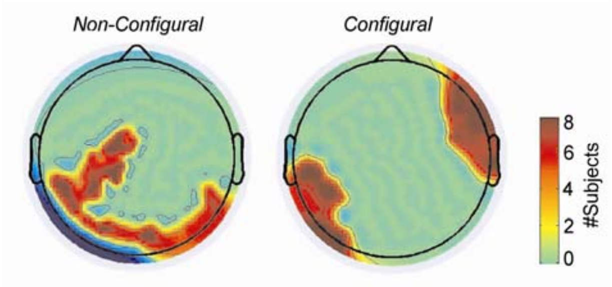

Figure S5. Within-subject phase-locking of reactivation time points to theta (6Hz). Results of the permutation analyses for those sensors included in the clusters identified in the cluster-based permutation analyses (see *Cluster-based non-parametric permutation test* section in the Supplemental Data). The colour scale in the topographic plots denotes the number of subjects where theta phase-locking of reactivations exceeded an alpha level of 0.05. Note that intermediate and blue colours are a mere product of the plotting interpolation function in the present figure.

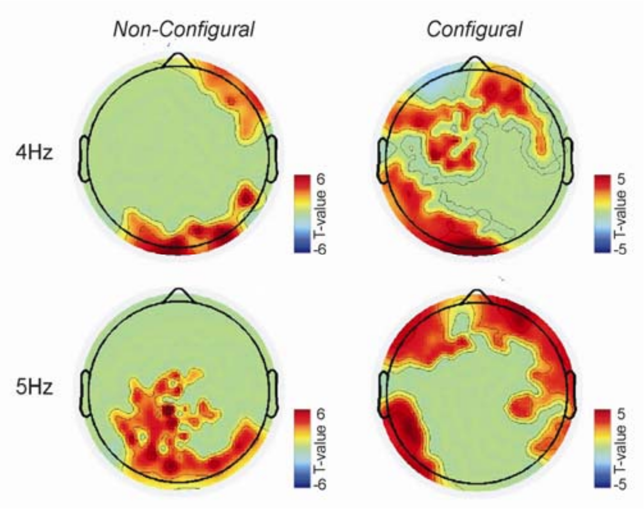

Figure S6. Theta phase-locking of reactivations at 4 and 5 Hz. Red areas indicate sensors where theta (4 Hz and 5 Hz) phase-coupling of category-specific reactivations during maintenance in *nonconfigural* and *configural* conditions was stronger ($p<0.05$) than in the *control* task. Only sensors clustered based on adjacency criteria (two sensors) are displayed. Note that finding similar PLV sensor distributions between the two frequencies could also be driven by aspects such as frequency resolution in the wavelet-based analyses or due to between-subject variability in the frequency PLV data. Also, note that intermediate and blue colours are a mere product of the plotting interpolation function in the present figure.

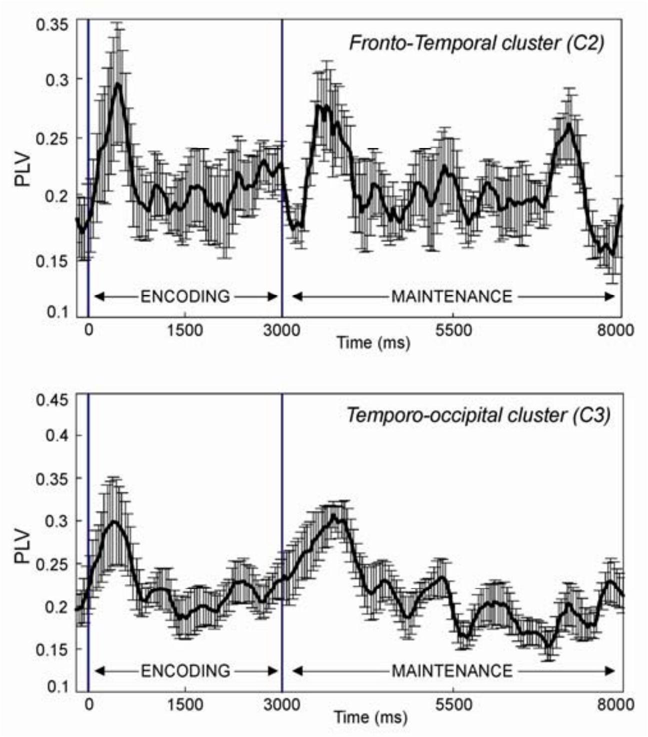

Figure S7. Averaged theta (6Hz) PLV from sensors included in the two sensor clusters corrected for multiple comparisons shown in Figure S4. PLV analysis was performed at each time point during encoding (3 sec) and maintenance (5 sec) periods regardless of the reactivation vector. 'This PLV analysis measures phase locking of theta activity between trials. Both sensor clusters showed an increase phase-reset at the onset of the delay maintenance period. The frontotemporal cluster also showed a phase-reset towards the end of the delay period.
